# Teaching stool form recognition in primary school: multimedia versus paper-based instruction using the Bristol stool form scale

**DOI:** 10.3389/fpubh.2026.1893172

**Published:** 2026-07-06

**Authors:** Chia-Jen Lee, Yung-Jen Yang, Ying-Jyun Shih, Jiun-Yi Wang

**Affiliations:** 1 Department of Healthcare Administration, College of Medical and Health Science, Asia University, Taichung, Taiwan; 2 Shuitsanlin Elementary School, Yunlin County, Taiwan; 3 Department of General Psychiatry, Tsaotun Psychiatric Center, Ministry of Health and Welfare, Tsaotun Township, Nan-Tou County, Taiwan; 4 Department of Nursing, Tsaotun Psychiatric Center, Ministry of Health and Welfare, Caotun, Nan-Tou County, Taiwan; 5 Department of Medical Research, China Medical University Hospital, China Medical University, Taichung, Taiwan

**Keywords:** Bristol stool forms scale, constipation, multimedia, paper-based, primary school students

## Abstract

**Background:**

Recognizing stool forms is essential for improving constipation awareness and promoting bowel health in children. This study aimed to evaluate primary school students’ ability to identify stool forms using the Bristol Stool Form Scale (BSFS).

**Methods:**

A quasi-experimental study was conducted between June 8 and 12, 2020, in a public primary school in Yunlin County, Taiwan. Students in grades 3 to 6 were enrolled and assigned to either a multimedia or a paper-based instructional condition. The multimedia learning group received pre-recorded audio-visual instruction, whereas those in the paper-based group engaged in self-learning using BSFS cards with textual explanations. All participants subsequently completed a 10-item stool-form identification test, and weighted scoring was applied to account for adjacent-type misclassification.

**Results:**

A total of 190 students were included in the final analysis (multimedia: *n* = 97; paper-based: *n* = 93). The mean weighted score (out of 10) was 8.64 ± 1.29 in the multimedia group and 8.91 ± 1.43 in the paper-based group, with no statistically significant difference between the groups (*p* = 0.165). Lower accuracy was observed for constipation-related stool types and mixed forms.

**Conclusion:**

Primary school students in grades 3 to 6 demonstrated high levels of stool-form recognition using the BSFS after brief structured instruction. No significant differences were observed between multimedia and paper-based instructional modalities. These findings suggest that either approach may be feasibly implemented in school-based health education.

## Introduction

Childhood constipation is a common health concern worldwide, affecting approximately 12.06% of school-aged children, with a substantial variation from 0.7 to 31.4% across different regions and settings (1). Persistent constipation in childhood may continue to adulthood and is associated with physical discomfort, lower quality of life, and increased healthcare utilization (2). Early recognition of abnormal bowel patterns is therefore an essential component of school-based health education and preventive care.

Health literacy in children requires appropriate instructional tools. For gastrointestinal health, the Bristol Stool Form Scale (BSFS) is a visual classification system consisting of seven stool types with brief descriptions and has been widely used in clinical and research settings (3, 4). Being translated to many languages, BSFS still demonstrates good reliability and validity across various studies (5, 6). Despite BSFS was originally developed based on the adult population, there have been advocates to use in children because of its ease to classify stool from visually. The 2024 ESPGHAN/NASPGHAN guidelines have recommended using BSFS as a tool for assessing pediatric stool form to enhance the objectivity of evaluation (7, 8). Because of its pictorial format, the BSFS may serve as a practical educational tool for children to learn stool consistency and bowel health.

Considering the usability in children, Chumpitazi et al. have proposed the modified Bristol Stool Form Scale for Children (mBSFS-C) with reduced number of stool categories from 7 to 5 to enhance usability (9). Subsequent studies have demonstrated the mBSFS-C to be a reliable and valid tool for children (10, 11), but there were also concerns regarding its sensitivity to the changes in stool consistency and its alignment with the Rome III criteria (7, 12).

Despite increased emphasis on bowel health education, research on how instructional modality influences children’s acquisition of stool-form recognition skills has been lacking. Both paper-based self-learning and multimedia instruction are two commonly used educational approaches in school settings, and the strengths and weaknesses of both modalities have been widely discussed (13, 14). In brief, paper-based learning may facilitate deeper processing and spatial organization of information (15, 16). In contrast, by integrates narration and visual cues, multimedia learning may enhance engagement (17, 18) but increase cognitive load (16–19). Some studies further suggest that sex and grade might play an influential role in learning outcomes in these contexts (20). Taken together, evidence comparing these modalities among primary school students has yielded mixed results.

To date, few studies have specifically compared the modalities in teaching stool-form recognition using the original seven-type BSFS in Asian primary school students. Moreover, limited research has addressed the misclassification patterns of recognizing types. Therefore, the present study aimed to (1) evaluate primary school students’ ability in identifying stool forms after structured instruction using the BSFS, (2) compare learning outcomes between multimedia and paper-based modalities, and (3) explore potential differences by sex and grade levels.

## Methods

### Study design and participants

This quasi-experimental study was conducted between June 8 and 12, 2020, in a public primary school in Yunlin County, Taiwan. Students from grades 3 to 6 in regular classes were eligible to participate, with each grade comprising two or three classes. This study was approved by the Institutional Review Board of Jen-Ai Hospital (IRB no. JA-109-44), and written informed consent was obtained from parents and children in advance.

Classes were used as the allocation unit to facilitate implementation within the school setting and minimize disruption to routine classroom activities. Instruction and assessment were scheduled according to the availability of individual classes. Within each grade, classes were assigned to either the multimedia or paper-based learning condition. When two classes were available within a grade, each class was randomly assigned to one instructional condition. When three classes were present, two classes were randomly selected for participation, and students in the remaining class were allocated according to pre-generated random numbers to balance group size. The allocation sequence was generated by the researcher (the first author) prior to data collection. Because the study was conducted within an existing school setting and was subject to administrative constraints, complete cluster randomization was not feasible.

Of 211 eligible students, 4 receiving resource-class services and 17 being absent on the assessment day were excluded. The final analytic sample included 190 students, with 97 in the multimedia group and 93 in the paper-based group.

Because all eligible students in grades 3–6 were invited to participate, the target sample size was determined by the available student population rather than a prior sample size calculation. Based on the available sample size (97 + 93), the study would have 80% power to detect a moderate effect size of 0.4 at *α* = 0.05.

### Instructional procedures

The BSFS used in this study consisted of seven stool types with corresponding visual images and textual descriptions. The translated materials were reviewed by an expert panel comprising two professors in healthcare and nursing, two physicians specializing in family medicine and gastroenterology, and one clinical nurse. The panel evaluated the appropriateness of the translated descriptions and their consistency with the original BSFS. The content validity index (CVI) for both dimensions exceeded 0.80 in the present study, which demonstrated satisfied content validity.

Both groups received structured instruction based on the original seven-type BSFS. In paper-based group, students read the printed BSFS cards (two A4 pages) containing images and corresponding textual descriptions for 10 min without discussion or teachers’ explanation. In multimedia group, students watched a 15-min pre-recorded slideshow presentation including BSFS images, textual descriptions, and narration. Within the 15-min presentation, the final section consisted of 10 review examples designed to reinforce previously presented BSFS content rather than introduce additional information.

To reduce classification ambiguity, both instructional materials included standardized guidance stating that when multiple stool characteristics were present, classification should be based on the most prominent or initial visual form.

### Assessment

A stool-form identification test was developed for this study to assess students’ ability to classify stool types using the BSFS. To minimize potential discomfort among children, all test items were presented using stylized illustrated images rather than real stool photographs. The initial item pool consisted of 15 images derived from a combination of BSFS materials, published sources, and researcher-developed illustrations.

In the first stage of development, two experts reviewed the preliminary items and recommended the removal of three images and revision of several researcher-developed illustrations to improve visual quality and realism, like color shading. The revised item pool was subsequently evaluated by the aforementioned panel of five experts. The experts assessed the appropriateness of the images and their consistency with the intended BSFS categories. Based on their recommendations, 10 items were retained for the final assessment. Eventually, the test covered all seven BSFS types, including additional items for Types 4 and 5 and one mixed-type item (Types 2–3). Immediately following instruction, students completed this 10-item stool-form identification test using illustrated images projected on screen.

Given the ordinal structure of the BSFS and common adjacent-type misclassification, we adopted a weighted scoring system in which correct response was defined to be 1 point, adjacent type to be 0.5 points, and non-adjacent type to be 0 points. Such a partial credit was intended to reflect partial understanding and reduce loss of information associated with strictly dichotomous scoring. The scoring scheme was prespecified and the total scores ranged from 0 to 10, with higher scores indicating better stool-form identification ability.

### Data analysis

Descriptive statistics were calculated for demographic characteristics and test scores. Independent samples t-tests were used to compare mean scores between instructional groups. Chi-square tests assessed baseline equivalence. Exploratory subgroup analyses were conducted by sex and grade level. The significance level of all hypothesis testing was set at 0.05. Because allocation was conducted primarily at the classroom level, responses from students within the same class may not have been fully independent. Given the limited number of participating classes, clustering effects were not explicitly modeled and should be considered when interpreting the findings. Considering the limited sample size, the cognitive difference due to developmental stages (21), and enhancing statistic power, students were grouped into middle (grades 3–4) and upper grades (grades 5–6) for analysis rather than being analyzed by individual grade.

## Results

**Figure 1:**
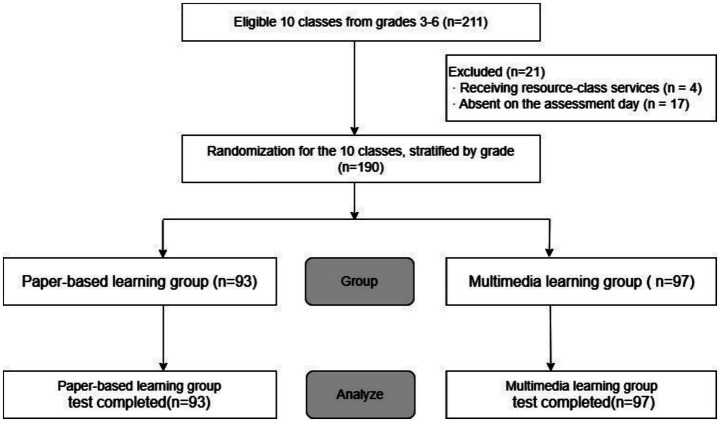
Flowchart of the enrollment.

A total of 211 students were initially enrolled from the selected classes, and 21 students were excluded from the study with reasons shown on Figure 1. The final analytic sample comprising 190 students, including 97 (51.1%) in the multimedia group and 93 (48.9%) in the paper-based group.

More boys were included in both groups with 60.8% in the multimedia group and 52.7% in the paper-based group, while more upper-grade students were also in both groups. However, no significant difference was observed between the two groups in terms of sex and grade. (Table 1).

**Table 1:** Characteristics of study participants.

Variables	Multimedia group	paper-based group	*p*
	*n* (%)	*n* (%)	
Gender			0.258
Female	38 (39.2)	44 (47.3)	
Male	59 (60.8)	49 (52.7)	
Grade			0.828
3–4	34 (35.1)	34 (36.6)	
5–6	63 (64.9)	59 (63.4)	

Results regarding the three aims of the present study were shown in Table 2, First, primary school students in middle and upper grades overall demonstrated high ability in identifying stool forms following brief structured instruction. The mean raw score of the stool-form identification test was slightly lower in the multimedia group than in the paper-based group [mean (M) = 8.16, standard deviation (SD) = 1.53 vs. M = 8.45, SD = 1.86]. However, such a difference was not remarkable (a trivial to small effect size of −0.17) and not statistically significant. Similar results were observed for the weighted scores (M = 8.64, SD = 1.29 vs. M = 8.91, SD = 1.43; effect size = −0.20), suggesting no significant difference in the achieved level of performance between multimedia and paper-based modalities.

**Table 2:** Test scores for the multimedia group and paper-based group.

Test results	multimedia group (*n* = 97)		paper-based group (*n* = 93)		95%CI of mean difference	*p*	Effect size
	M	SD	M	SD			
Total							
Raw score	8.16	1.53	8.45	1.86	(−0.75, 0.27)	0.245	−0.17
Weighted score	8.64	1.29	8.91	1.43	(−0.65, 0.11)	0.165	−0.20
Sex (weighted score)							
Female	8.28	1.65	9.35	0.87	(−1.64, −0.50)	0.001^**^	−0.81
Male	8.87	0.94	8.52	1.70	(−0.16, 0.86)	0.198	0.26
Grade (weighted score)							
3–4	8.54	1.18	8.75	1.68	(−0.49, 0.07)	0.560	−0.15
5–6	8.69	1.35	9.01	1.26	(−0.79, 0.15)	0.183	−0.25

^**^p < 0.01. M, mean; SD, standard deviation.

Moreover, subgroup analyses by sex indicated that, girls in the multimedia group had a significantly lower mean weighted score than those in the paper-based group (M = 8.28, SD = 1.65 vs. M = 9.35, SD = 0.87, *p* = 0.001), which showed a large effect size of −0.81. In contrast, boys in the multimedia group had a slightly higher mean weighted score than those in the paper-based group (M = 8.87, SD = 0.94 vs. M = 8.52, SD = 1.70, *p* = 0.198), but the difference was not statistically significant.

Within-group comparisons further showed sex differences under the same instructional modality. In the multimedia group, boys had higher mean weighted scores than girls (8.87 vs. 8.28, *p* = 0.048), while girls outperformed boys in the paper-based group (9.35 vs. 8.52, *p* = 0.003).

When stratified by grade level, mean weighted scores in the multimedia group were slightly lower than those in the paper-based group for both middle-grade students (M = 8.54, SD = 1.18 vs. M = 8.75, SD = 1.68; effect size = −0.15) and upper-grade students (M = 8.69, SD = 1.35 vs. M = 9.01, SD = 1.26; effect size = −0.25). However, these differences were not statistically significant.

**Figure 2:**
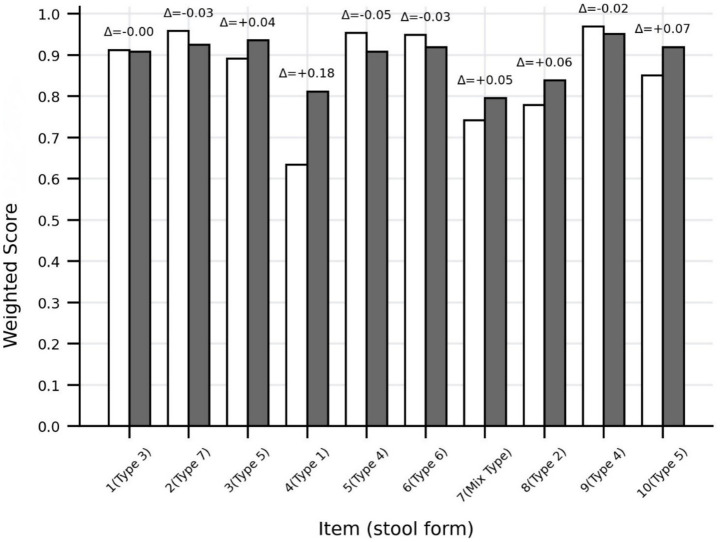
Mean weighted scores of each item in the test between the multimedia (white bar) and paper-based (gray bar) groups (the delta shows the mean difference).

Item-level analysis of the 10-item test (Figure 2) showed that, in the multimedia group, the lowest weighted scores were observed for question 4 (Type 1; 0.63), followed by question 7 (mixed type; 0.74) and question 9 (Type 2; 0.78). In the paper-based group, the lowest scores were observed for question 7 (mixed type; 0.80), followed by question 4 (Type 1; 0.81) and question 9 (Type 2; 0.84). Weighted scores for all other items in both groups were above 0.85.

## Discussion

The present study examined whether primary school students could demonstrate stool-form recognition through brief structured instruction and whether instructional modalities were associated with different levels of performance. Our results showed an overall accuracy of over 80% in both groups and suggested that students in grades 3–6 can achieve high levels of stool-form recognition using the BSFS after short educational exposure through both instructive modalities. However, no statistically significant difference was observed between multimedia and paper-based modalities.

### The accuracy of stool-form recognition

The mean raw scores of the stool-form test in the multimedia and paper-based groups were 8.16 and 8.45, respectively, and the corresponding weighted scores were 8.64 and 8.91. The overall high accuracy observed in both groups suggests that primary school students were able to achieve a high level of stool-form recognition following structured instruction, which may have implications for supporting children’s bowel health literacy.

Previous studies have reported that children’s accuracy in using the original BSFS is approximately 71.8% (11), with particular difficulty in distinguishing borderline categories, such as Types 2 versus 3 and Types 5 versus 6 (22). To address these challenges, modified versions such as the mBSFS-C have been developed, achieving improved accuracy rates ranging from 78 to 88% (10, 11, 22). However, our study found that, with structured instructional support, the original BSFS can achieve a comparable performance. The observed mean scores in both groups suggested that the difficulty associated with the original scale is not insuperable, and that learning outcomes might largely depend on the provision of adequate learning support. These results further indicated that supporting stool-form recognition could be achieved more on well-designed instructional support than on developing or modifying the scale or employing the specific delivery modality.

At the item level, lower accuracy was observed for constipation-related types (Types 1 and 2). Students frequently misclassified Type 1 as Type 5 and had difficulty distinguishing between Types 2 and 3. Such phenomena were consistent with the findings from previous studies in children (10, 23). Prior research has also shown that children tend to perform better when identifying more prototypical or extreme stool forms but make more errors in visually similar or borderline categories (10, 11, 22). This pattern may be explained by children’s reliance on overall contour rather than more subtle features such as surface texture or segmentation (24, 25). In addition, the painted images of lumpy stools (Types 1 and 5) might not adequately convey differences in texture or edge definition and led to misclassification. Comparable classifying patterns have been reported in adult populations, where Types 4 and 7 are most accurately identified, while Types 1 and 5 are more frequently confused (14, 23, 26). Overall, the error patterns observed in this study are consistent with those reported in adult research. In the present study, mixed forms proved particularly challenging for students with the average weighted score for mixed types in the multimedia group was only 0.74. This finding was consistent with previous findings in the adult population which suggested the recognition accuracy for mixed types usually fell below 80% (12). Despite these inherent difficulties, students still demonstrated high overall recognition accuracy following instruction.

Taken together, these findings suggest that structured instruction may be a useful approach for supporting stool-form recognition among primary school students. Health education programs may place greater emphasis on distinguishing constipation-related stool types and providing explicit guidance for interpreting mixed forms stool to support accurate recognition.

### Instructional modalities and health education implications

Although multimedia learning integrated narration and visual cues, and paper-based learning might promote deeper processing, the present findings indicated that both approaches resulted in similarly levels of performance for this visually driven categorization task. This aligned with educational research finding that learning outcomes often depended more on instructional design and content clarity than on the delivery medium (27).

From a school health education perspective, this flexibility across different delivery media is advantageous. Schools with limited technological resources can rely on paper-based materials while still achieving comparable levels of student performance, whereas multimedia tools may enhance engagement in resource abundant areas.

It is worth to note that although students demonstrated high levels of stool-form recognition in the assessment, the study did not evaluate whether this knowledge could be transferred to real-life settings, such as recognizing their own stool forms or reporting bowel habits more accurately. Therefore, future studies could be conducted to examine the retention of stool-form recognition skills and their applicability in everyday contexts.

### Sex and grade-level findings

The subgroup analyses revealed sex differences in modalities, with girls performing significantly better than boys in the paper-based group. This might be related to the differences in coding and memory processes in primary school girls (28). Although the overall performance between the two modalities were not significantly different, our findings suggest that individual learner characteristics still might interact with instructional format. However, we should emphasize the exploratory nature of such a result, especially because cognitive style and learning preferences were not directly measured, these interpretations should be considered hypothesis-generating rather than confirmatory, and deserved future study.

Grade-level comparisons showed no significant differences between middle- and upper-grade students in either overall or specific modality, and this finding suggested that stool-form recognition ability could be sufficiently developed as early as the students in grade 3. Other studies have similar findings (22).

### Strengths and limitations

This study contributed to the school health literature by applying a weighted scoring approach to capture partial understanding and adjacent misclassification patterns. The study design also reflects realistic classroom implementation. To the best of our knowledge, this was the first study that explored children’s ability to identify all seven stool forms based on the BSFS in the East Asian region.

Several limitations should be acknowledged. First, it was conducted in a single school, which may limit the representativeness of the sample and the generalizability of the findings. Second, no baseline assessment nor non-instructional comparison group was included; therefore, changes in stool-form recognition following instruction could not be directly evaluated. Third, the two instructional conditions were not fully equivalent in terms of delivery format and instructional exposure. In addition, allocation was conducted primarily at the classroom level, and clustering effects were not explicitly modeled in the statistical analyses. Fourth, the assessment measured immediate post-instruction performance and did not evaluate long-term retention. The observed accuracy may therefore have been influenced by short-term memory and could overestimate practical classification ability. Fifth, although the assessment images underwent expert review, further evaluation of their psychometric properties is warranted. The study used illustrated images rather than real photographs, which may not fully reflect real-world recognition. However, this approach was considered appropriate because real stool photographs may cause psychological discomfort and reduce participation among children. Finally, a potential ceiling effect should be considered, as both groups achieved relatively high scores, which may have reduced the ability to detect differences between instructional modalities.

## Conclusion

Primary school students in grades 3 to 6 demonstrated high levels of stool-form recognition using the BSFS following brief structured instruction. As no significant differences were observed between multimedia and paper-based instructional modalities, either approach may be feasibly implemented according to available educational resources and classroom contexts. Future school health programs may place greater emphasis on helping students distinguish different stool types and should consider evaluating the long-term retention and behavioral implications of stool-form recognition.

## Data Availability

The raw data supporting the conclusions of this article will be made available by the authors, without undue reservation.
